# Changes in physical activity and modelled cardiovascular risk following diagnosis of diabetes: 1-year results from the ADDITION-Cambridge trial cohort

**DOI:** 10.1111/j.1464-5491.2012.03765.x

**Published:** 2013-02

**Authors:** A Barakat, K M Williams, A T Prevost, A-L Kinmonth, N J Wareham, S J Griffin, R K Simmons

**Affiliations:** 1 Department of Health Sciences and EMGO Institute for Health and Care Research, VU University Medical CentreAmsterdam, The Netherlands; 2 MRC Epidemiology UnitCambridge; 3 General Practice and Primary Care Research Unit, Institute of Public HealthCambridge; 4 King's College London, Department of Primary Care and Public Health SciencesLondon, UK

## Abstract

**Aims:**

To describe change in physical activity over 1 year and associations with change in cardiovascular disease risk factors in a population with screen-detected Type 2 diabetes.

**Methods:**

Eight hundred and sixty-seven individuals with screen-detected diabetes underwent measurement of self-reported physical activity, cardiovascular disease risk factors and modelled cardiovascular disease risk at baseline and 1 year (*n* = 736) in the ADDITION-Cambridge trial. Multiple linear regression was used to quantify the association between change in different physical activity domains and cardiovascular disease risk factors at 1 year.

**Results:**

There was no change in self-reported physical activity over 12 months. Even relatively large changes in physical activity were associated with relatively small changes in cardiovascular disease risk factors after allowing for changes in self-reported medication and diet. For every 30 metabolic equivalent-h increase in recreational activity (equivalent to 10 h/brisk walking/week), there was an average reduction of 0.1% in HbA_1c_ in men (95% CI −0.15 to −0.01, *P* = 0.021) and an average reduction of 2 mmHg in systolic blood pressure in women (95% CI −4.0 to −0.05, *P* = 0.045).

**Conclusions:**

Few associations were observed between change in different physical activity domains and cardiovascular disease risk factors in this trial cohort. Cardiovascular disease risk reduction appeared to be driven largely by factors other than changes in self-reported physical activity in the first year following diagnosis.

## Introduction

Following diagnosis of Type 2 diabetes, patients are advised about the importance of physical activity for weight loss and for controlling blood glucose, blood pressure and blood lipid levels. There is good evidence that exercise training has a beneficial effect on cardiovascular risk factors in people with established diabetes [1–5]. Most research on physical activity recommendations in diabetes focuses on individuals with clinically diagnosed and/or well-established disease. Given that population screening for diabetes has been recommended by several national organizations and the National Health Service (NHS) currently includes assessment of risk of diabetes in its Health Checks programme [6], more individuals will be found earlier in the disease trajectory, where there is little current evidence for physical activity recommendations. Indeed, the National Institute for Health and Clinical Excellence (NICE) do not offer explicit guidance on exercise type, frequency or duration in Type 2 diabetes. Furthering our understanding of cardiovascular disease risk reduction in individuals with screen-detected diabetes will thus allow improvement in diabetes care and appropriate targeting of resources.

ADDITION-Cambridge is a primary care-based study of screening for Type 2 diabetes followed by a pragmatic cluster randomized controlled trial comparing intensive multifactorial treatment with routine care in patients with screen-detected diabetes. We aimed (1) to describe change in different physical activity domains and modelled cardiovascular disease risk over 1 year and (2) to explore whether change in physical activity was associated with a reduction in modelled cardiovascular disease risk in this screen-detected population.

## Patients and methods

The design and rationale for ADDITION-Cambridge have previously been reported [7]. In brief, 49 general practice surgeries in the Eastern region of England recruited patients through a stepwise screening programme. Individuals were eligible for screening if they were registered with one of the participating general practices, were aged 40–69 years, not known to have diabetes and with a diabetes risk score of > 0.17 (corresponding to the top 25% of the population distribution [8]). Exclusion criteria included pregnancy, lactation, an illness with a life expectancy of less than 12 months or a psychiatric disorder that might invalidate informed consent. Thus, 33 539 eligible participants were invited to take part in the screening programme [9]. World Health Organization (WHO) criteria were used to diagnose diabetes [10]. In total, 867 individuals with screen-detected diabetes agreed to participate and provided written informed consent. Ethical approval was granted by the Eastern Multi-Regional Ethics Committee (reference 02/5/54).

Participants were managed according to the treatment regimen to which their practice was allocated: intensive treatment or routine care. The intensification of diabetes management was achieved through the addition of a number of features to existing diabetes care [7,11], alongside lifestyle advice concerning diet, physical activity and tobacco consumption, and a stepwise target-led drug treatment regime to reduce hyperglycaemia, blood pressure, hyperlipidaemia and microalbuminuria. Routine care practices followed current UK national guidelines for diabetes management [12–14].

### Measurement and outcomes

Baseline and 1-year health assessments included physiological and anthropometric measurements, venesection and the completion of questionnaires [7]. Modelled 10-year risk of cardiovascular disease was calculated using the UK Prospective Diabetes Study (UKPDS) risk engine (version 3.0) [15]. Data on physical activity were collected using the European Prospective Investigation into Cancer (EPIC)-Norfolk physical activity questionnaire (EPAQ-2 version 4.0), which has previously been validated using objective heart rate monitoring [16]. This self-report questionnaire is designed to measure domain-specific physical activity in the past year and asks about physical activity patterns in and around the house, activity at work, travel to work and recreational physical activity. Vigorous physical activity was calculated using duration, type and frequency of exercise collected in the recreational physical activity section.

### Statistical analyses

Baseline and follow-up characteristics were summarized separately in men and women. Multiple linear regression models were used to describe the association between change in physical activity and cardiovascular risk factors and modelled cardiovascular risk at 1 year; results are reported as standardised β-coefficients. All models were run separately by sex and adjusted for baseline physical activity behaviour, age, randomization group, change in smoking status, change in calorie intake and relevant medication. Data were analysed using SPSS (version 18.0; SPSS Inc., Chicago, IL, USA).

## Results

Baseline characteristics of ADDITION-Cambridge participants with complete data for baseline and follow-up (*n* = 736) are presented separately by gender in Table 1. Non-attenders to the follow-up health assessment reported lower levels of baseline recreational physical activity and were more likely to have experienced a previous heart attack or stroke than attenders. For all other characteristics there were no significant differences between attenders and non-attenders (data not shown).

**Table 1:** Characteristics of ADDITION-Cambridge participants with complete data at baseline and 1-year follow-up (*n* = 736)

	Men (*n* = 453)				Women (*n* = 283)			
	
	*n*	Baseline	Follow-up	*P*-value*	*n*	Baseline	Follow-up	*P*-value*
Age, years	453	60.3 (7.5)	—	—	283	62.3 (6.3)	—	—
Age finished full-time education, %								
< 16 years	453	45.2	—	—	283	52.0	—	—
16–18 years		42.4				38.8		
> 18 years		12.4				9.3		
Ethnicity, %								
White	452	97.6	—	—	283	95.8	—	—
Asian		2.2				3.5		
Previous heart attack, % yes	452	10.9	11.4	0.65	283	2.5	3.6	0.56
Previous stoke, % yes	452	3.8	4.6	0.32	283	3.9	5.5	0.13
Weight, kg	448	98.6 (17.3)	95.4 (16.8)	< 0.001	282	88.2 (15.9)	84.0 (15.8)	< 0.001
Waist circumference, cm	451	114.2 (13.0)	111.4 (12.9)	< 0.001	282	107.5 (13.0)	103.9 (13.0)	< 0.001
BMI, kg/m^2^	448	32.6 (5.2)	31.7 (5.1)	< 0.001	281	34.5 (5.9)	33.0 ( 5.9)	< 0.001
Alcohol, units/week	438	10.0 (12.3)	8.9 (11.3)	0.003	269	3.6 (6.1)	3.1 (5.1)	< 0.001
Smoking status, %								
Non-smoker/former	445	81.0	82.7	0.23	279	86.2	88.9	0.01
Current smoker		19.0	17.3			13.8	11.1	
Total energy intake, kcal/day	440	2014.8 (708.6)	1731.4 (527.9)	< 0.001	276	1797.7 (625.5)	1631.7 (603.8)	< 0.001
Self-reported physical activity								
Home (MET h/week)	433	29.7 (28.6)	31.5 (25.7)	0.15	269	59.9 (30.4)	59.9 (33.0)	0.97
Work (MET h/week)†	204	104.0 (55.6)	102.3 (59.2)	0.55	76	62.5 (41.5)	66.4 (49.1)	0.34
Recreational (MET h/week)	413	33.9 (40.2)	37.8 (40.9)	0.055	271	22.3 (30.4)	20.4 (22.6)	0.31
Vigorous (h/week)	451	0.41 (1.7)	0.59 (1.6)	0.080	280	0.24 (0.7)	0.32 (0.8)	0.098
Prescribed medication, % yes								
Lipid lowering medication	447	25.4	57.1	< 0.001	282	20.1	52.8	< 0.001
Glucose lowering medication	—	—	31.1	—	—	—	28.7	—
Anti-hypertensive medication	447	51.9	64.4	< 0.001	282	61.1	69.9	< 0.001
Cardiovascular risk factors								
Total cholesterol, mmol/l	443	5.3 (1.1)	4.4 (1.0)	< 0.001	274	5.6 (1.2)	4.7 (0.8)	< 0.001
Systolic blood pressure, mmHg	451	143.2 (19.9)	138.3 (18.2)	< 0.001	280	139.5 (19.8)	132.9 (18.7)	< 0.001
HbA_1c_, mmol/mol	440	57 (N/A)§	48 (N/A)§	< 0.001	269	55 (N/A)§	48 (N/A)§	< 0.001
HbA_1c,_%	440	7.4 (1.8)	6.5 (0.9)	< 0.001	269	7.2 (1.6)	6.5 (0.8)	< 0.001
Modelled 10-year cardiovascular disease risk‡	423	0.36 (0.1)	0.31 (0.1)	< 0.001	255	0.23 (0.1)	0.18 (0.09)	< 0.001

All values are means (SD) unless otherwise indicated.

* McNemar’s test for categorical variables and paired *t*-test for continuous variables.

† Including only those participants who had a job at baseline and 1 year.

‡ Calculated using the UK Prospective Diabetes Study (UKPDS) risk engine (version 3), excluding participants who experienced a previous heart attack or stroke.

§ It is not possible to calculate International Federation of Clinical Chemistry and Laboratory Medicine (IFCC) values for low HbA1c values.

The sample size was at least 413 for men and 255 for women for all variables, with the exception of work (self-reported physical activity) (men = 204; women = 76)

MET, metabolic equivalent; N/A, not available.

### Change in cardiovascular disease risk factors, modelled cardiovascular disease risk and physical activity

As shown in Table 1, for both men and women, there were significant reductions in anthropometric and biochemical risk factors, alongside increases in the prescription of cardio-protective medication and improvements in calorie intake, alcohol consumption and smoking status.

In women, there was no change in home, work or recreational physical activity between baseline and follow-up. There was a small non-significant increase in vigorous physical activity (0.08 h/week). For men, there were small non-significant increases in recreational (3.9 metabolic equivalent h/week) and vigorous physical activity (0.18 h/week). Although mean change in self-reported physical activity from baseline to follow-up was non-significant, the standard deviations for physical activity change were very large, indicating that some individuals reported large increases or reductions in their physical activity behaviour.

### Association between change in physical activity, cardiovascular disease risk factors and modelled cardiovascular disease risk

As shown in Table 2, for men, an increase in recreational physical activity over 1 year was independently associated with a reduction in HbA_1c_ [β (95% CI); −0.093 (−0.17 to −0.014), *P* = 0.021], while in women an increase in recreational physical activity was independently associated with a reduction in systolic blood pressure [β (95% CI); −2.32 (−4.60 to −0.051, *P* = 0.045]. There were no significant associations between change in any other physical activity domain, cardiovascular disease risk factors and modelled cardiovascular disease risk at 1 year. Although the β-coefficients were largely in the expected direction of effect, the coefficients were small and not statistically significant.

**Table 2:** Crude and adjusted associations between change in physical activity, cardiovascular disease risk factors and modelled cardiovascular disesae risk at follow-up in the ADDITION-Cambridge trial cohort

Women													

Outcome		Standardized β-coefficient*											

		Total cholesterol	95% CI	*P*-value	Systolic blood pressure	95% CI	*P*-value	HbA_1c_	95% CI	*P*-value	Modelled cardiovascular disease risk§	95% CI	*P*-value	
Δ home physical activity	Crude	−0.042	−0.15 to 0.063	0.43	−1.42	−3.63 to 0.98	0.21	−0.044	−0.14 to 0.049	0.35	−0.004	−0.014 to 0.006	0.39
(MET h/week)	Adjusted†	−0.030	−0.11 to 0.056	0.49	−1.50	−3.41 to 0.41	0.12	−0.030	−0.11 to 0.048	0.45	−0.003	−0.010 to 0.003	0.33
Δ work physical activity‡	Crude	−0.17	−0.42 to 0.075	0.17	0.44	−5.26 to 6.14	0.88	−0.026	−0.23 to 0.18	0.80	−0.006	−0.023 to 0.011	0.48
(MET h/week)	Adjusted†	−0.083	−0.28 to 0.12	0.41	1.52	−3.58 to 6.62	0.55	0.053	−0.15 to 0.26	0.61	0.003	−0.007 to 0.012	0.56
Δ recreational physicalactivity	Crude	−0.009	−0.03 to 0.12	0.89	−2.01	−4.69 to 0.68	0.14	0.027	−0.083 to 0.14	0.93	−0.006	−0.018 to 0.006	0.29
(MET h/week)	Adjusted†	−0.029	−0.13 to 0.076	0.59	−2.32	−4.60 to −0.05	0.045	0.022	−0.072 to 0.12	0.65	−0.005	−0.013 to 0.003	0.22
Δ vigorous physical activity	Crude	−0.14	−0.39 to 0.10	0.25	−4.39	−9.44 to 0.66	0.088	0.020	−0.19 to 0.24	0.85	−0.016	−0.039 to 0.007	0.16
(h/week)	Adjusted†	−0.15	−0.34 to 0.050	0.14	−2.49	−6.83 to 1.85	0.26	0.023	−0.16 to 0.21	0.80	−0.003	−0.018 to 0.012	0.71

Men													

Outcome		Standardized β-coefficient*											

		Total cholesterol	95% CI	*P*-value	Systolic blood pressure	95% CI	*P*-value	HbA_1c_	95% CI	*P*-value	Modelled cardiovascular disease risk§	95% CI	*P*-value	
Δ home physical activity	Crude	−0.071	−0.17 to 0.029	0.16	−0.52	−2.29 to 1.26	0.57	−0.004	−0.094 to 0.086	0.93	−0.001	−0.013 to 0.011	0.87
(MET h/week)	Adjusted†	−0.021	−0.098 to 0.056	0.60	−0.84	−2.34 to 0.66	0.27	0.009	−0.075 to 0.093	0.83	−0.003	−0.010 to 0.005	0.48
Δ work physical activity ‡^c^	Crude	−0.055	−0.19 to 0.077	0.41	−2.55	−4.96 to −0.14	0.038	−0.11	−0.24 to 0.008	0.067	−0.012	−0.026 to 0.003	0.12
(MET h/week)	Adjusted†	−0.001	−0.093 to 0.090	0.98	−1.21	−3.17 to 0.76	0.23	−0.079	−0.19 to 0.037	0.18	−0.004	−0.013 to 0.005	0.40
Δ recreational physical activity	Crude	0.001	−0.089 to 0.091	0.98	0.16	−1.50 to 1.82	0.85	−0.085	−0.17 to 0.001	0.052	−0.012	−0.12 to 0.014	0.028
(MET h/week)	Adjusted†	0.012	−0.058 to 0.081	0.74	0.79	−0.62 to 2.20	0.27	−0.093	−0.17 to −0.014	0.021	−0.006	−0.013 to 0.001	0.11
Δ vigorous physical activity	Crude	0.069	−0.008 to 0.15	0.079	0.90	−0.48 to 2.29	0.20	−0.035	−0.11 to 0.036	0.33	0.003	−0.006 to 0.012	0.53
(h/week)	Adjusted†	0.029	−0.031 to 0.089	0.35	0.56	−0.60 to 1.73	0.34	−0.033	−0.099 to 0.033	0.33	0.001	−0.005 to 0.007	0.67

* For every standard deviation unit increase in [physical activity domain], there was an average [increase/decrease] of [XX] units in [cardiovascular disease risk factor].

† Adjusted for baseline physical activity behaviour, age, randomization group, change in smoking, change in calorie intake and change in medication use (total cholesterol adjusted for change in lipid-lowering medication, systolic blood pressure adjusted for change in anti-hypertensive medication, HbA1c adjusted for glucose-lowering medication at 1 year, and modelled cardiovascular disease risk adjusted for change in lipid-lowering, anti-hypertensive medication and glucose-lowering medication at 1 year).

‡ Includes only participants who had a job at baseline and after 1 year.

§ Calculated using the UK Prospective Diabetes Study (UKPDS) risk engine (version 3), excluding participants who experienced a previous heart attack or stroke.

MET, metabolic equivalent.

## Discussion

There was no change in self-reported physical activity over 12 months in a population of patients newly diagnosed with Type 2 diabetes in the East of England. There were significant decreases in cardiovascular disease risk factors and modelled cardiovascular disease risk. Increases in recreational physical activity were associated with a significant reduction in HbA_1c_ in men and systolic blood pressure in women. Changes in other physical activity domains were not associated with a reduction in modelled cardiovascular disease risk. Our finding suggests that more attention may need to be paid to the promotion of physical activity following diagnosis of diabetes. Reduction in modelled cardiovascular disease risk may be driven by factors other than self-reported physical activity in this screen-detected cohort. These include changes in medication and dietary behaviour [17].

Similar reductions in cardiovascular disease risk factors have been seen in patients with diabetes enrolled in lifestyle interventions. In the Italian Diabetes and Exercise Study (IDES) study [4], 606 sedentary individuals with diabetes and the metabolic syndrome were randomized to twice-weekly supervised aerobic and resistance training and structured exercise counselling, or counselling alone (control group) for 12 months. Increases in physical activity were associated with significant improvements in fitness, HbA_1c_, blood pressure, cholesterol, waist circumference and modelled coronary heart disease risk. Contrary to our findings, self-reported physical activity remained a significant driver of cardiovascular disease risk reduction in this cohort even after adjustment for statin treatment (the only medication that changed significantly in both groups). In the Early Activity in Diabetes (Early ACTID) trial, 593 recently diagnosed patients with diabetes were randomized to (1) usual care (control), (2) an intensive diet intervention or (3) an intensive diet intervention plus a pedometer-based activity programme [17]. After 12 months, there were significant improvements in glycaemic control, insulin resistance and bodyweight in both intervention groups compared to the control; however, the addition of the activity intervention conferred no extra benefit. Other studies examining behavioural change in patients with Type 2 diabetes tend to be small, of shorter duration and focus on individuals later in the disease trajectory.

It is possible that physical activity did not significantly increase over 1 year in the ADDITION-Cambridge cohort because of the nature of the physical activity intervention. Participants in the intensive treatment group were advised to increase their physical activity to reach a goal of 35 min of brisk walking every day. However, other physical activity studies in patients with diabetes have set more challenging goals; for example, a minimum of 150 min/week of progressive mixed training [4], as well as offering a higher number of counselling sessions [3]. Measures of change in physical activity were calculated from a self-report questionnaire, which may have been subject to error and bias. Physical activity might have increased a little after diagnosis, but changes over the time period covered by the EPIC-Norfolk physical activity questionnaire were not apparent. We may not therefore have captured the full extent of physical activity behaviour change. Finally, physical activity behaviour may not be a driver of reduction in risk factors in screen-detected individuals, who are earlier in the diabetes disease trajectory and for whom the initiation of a medication regime may be the biggest change in the first year following diagnosis.

## Strengths and limitations

Anthropometric and clinical measurements were undertaken by trained staff following standard operating procedures. We adjusted for both change in medication use and change in total calorie intake between baseline and 1 year, which might have impacted on cardiovascular disease risk at follow-up, and which other studies have not adjusted for [3,4]. The study is of larger size and longer duration than many studies in patients with diabetes, which are typically limited to less than 1 year. Extrapolation of our results to more deprived and ethnically diverse settings may be limited in light of the non-random recruitment of general practices from a single geographical region (Eastern England). We conducted multiple significance tests (> 20) between change in physical activity and cardiovascular disease risk factors which may have led to an increased risk of Type 1 errors. It is therefore unclear whether the few significant observations represent real or chance associations.

## Conclusions

Few associations were observed between change in physical activity, cardiovascular disease risk factors and modelled cardiovascular disease risk over 12 months in ADDITION-Cambridge. The observed reduction in cardiovascular disease risk factors may have been driven by factors other than physical activity in the first year following diagnosis. Physical activity has benefits beyond effects on cardiovascular disease risk factors at 1 year and therefore merits promotion in patients with diabetes.

### Funding sources

None.

### Competing interests

None declared.
